# Nominal Differences in Acute Symptom Presentation and Recovery Duration of Sport-Related Concussion Between Male and Female Collegiate Athletes in the PAC-12

**DOI:** 10.1186/s40798-024-00699-4

**Published:** 2024-04-02

**Authors:** Niki A. Konstantinides, Sean M. Murphy, Bridget M. Whelan, Kimberly G. Harmon, Sourav K. Poddar, Theresa D. Hernández, Rachel K. Rowe

**Affiliations:** 1 Department of Integrative Physiology, University of Colorado Boulder, UCB 354, Boulder, CO 80309 USA; 2 Cumberland Biological and Ecological Researchers, Longmont, CO USA; 3 Family Medicine, University of Washington, Seattle, WA USA; 4 Department of Family Medicine, University of Colorado Anschutz Medical Campus, Aurora, CO USA; 5 Psychology and Neuroscience (CU Boulder), Physical Medicine and Rehabilitation (CU Anschutz School of Medicine), Aurora, CO USA

**Keywords:** Clinical profiles, Sport-related concussion, Time-to-recovery, Return-to-play, Athletes

## Abstract

**Background:**

Sport-related concussion (SRC) is a heterogenous injury that often presents with varied symptoms and impairment. Recently, research has focused on identifying subtypes, or clinical profiles of concussion to be used in assessing and treating athletes with SRC. The purpose of this study was to investigate sex differences in clinical profiles, recovery duration, and initial symptom severity after SRC in a cohort of collegiate athletes in the Pacific-12 Conference (Pac-12).

**Methods:**

This prospective cohort study examined post-SRC symptoms, recovery, and return-to-play times using data from the Pac-12 CARE Affiliated Program and Pac-12 Health Analytics Program. Clinical profiles reported by student-athletes were defined by the number (> 50%) of specific symptoms frequently reported for each profile. Generalized linear mixed models were used to examine associations among sex, clinical profiles, time-to-recovery, and return-to-play times.

**Results:**

479 concussion incidents met inclusion criteria. The probabilities of initial presentation of each clinical profile, initial injury severity scores, and recovery times within a profile did not differ between sexes (*p* = 0.33–0.98). However, both males and females had > 0.75 probabilities of exhibiting cognitive and ocular profiles. Initial injury severity score was a strong nonlinear predictor of initial number of clinical profiles (*p* < 0.0001), which did not differ between sexes. The number of clinical profiles was also a nonlinear predictor of time-to-recovery (*p* = 0.03) and return-to-play times (*p* < 0.0001).

**Conclusions:**

Initial symptom severity was strongly predictive of the number of acute clinical profiles experienced post-SRC. As the number of clinical profiles increased, time-to-recovery and time to return-to-play also increased. Factors other than sex may be better associated with acute symptom presentation post-concussion as no sex differences were found in reported clinical profiles or recovery. Understanding the number and type of clinical profiles experienced post-SRC may help inform concussion diagnostics and management.

**Supplementary Information:**

The online version contains supplementary material available at 10.1186/s40798-024-00699-4.

## Key Points

- The number of clinical profiles reported, and initial symptom severity were associated with prolonged SRC recovery in athletes. There was also a high probability of collegiate athletes experiencing cognitive and ocular symptoms at acute concussion.
- No sex differences were found in reported clinical profiles or time-to-recovery and time to return-to-play.
- This is clinically important because identifying what characteristics of concussion may be associated with diagnosis and prolonged time-to-recovery and time to return-to-play can facilitate more targeted symptom management strategies after SRC.

**Supplementary Information:**

The online version contains supplementary material available at 10.1186/s40798-024-00699-4.

## Background

Gaining an understanding of the heterogeneity of symptom presentation and time-to-recovery from sport-related concussion (SRC) has received increasing interest in recent years [1–3]. Athletes exhibit different subtypes or clinical profiles of SRC, which tend to be associated with distinct sets of symptoms and recovery trajectories [3–7]. Identifying post-concussion clinical profiles and matching targeted and active treatments to those profiles may improve outcomes and shorten recovery times in athletes. Previous research found dependencies in symptom presentation [1] and evidence that symptoms/clinical profiles co-occur because of strong interrelatedness [8]. However, little is understood about how symptom presentation influences symptom resolution time, because the use of clinical profiles for diagnostics is still an emerging concept [3].

Previous analyses of symptom scales in collegiate athletes identified four distinct subdomains of concussion symptoms without significant symptom overlap: physical, cognitive, emotional, and sleep/fatigue [7, 9, 10]. In contrast, other studies categorized symptoms and functional impairments of SRC among six proposed clinical profiles, including: affective (anxiety/mood), fatigue, cognitive, headache/migraine, vestibular, and ocular; however, there is significant symptom overlap among these defined clinical profiles [3, 4]. Another challenge to determining clinical profiles experienced following concussion is that many athletes may present with more than one clinical profile [4, 6]. Although it has been recommended to prioritize each clinical profile as “primary, secondary, and tertiary,” overlapping symptoms between profiles further complicates this ranking process [6]. Although the connection between reported clinical profiles and time-to-recovery in collegiate athletes has yet to be explored, a strong association between specific symptoms, such as, irritability, difficulty concentrating, noise sensitivity, and dizziness reported after concussion and time to symptom resolution has been reported [1, 2].

Evidence indicates that, on average, females report significantly more symptoms and greater total symptom severity [11] after concussion than do males [12–16], but some studies found that recovery patterns [17, 18] and total number of symptoms [18, 19] were similar between sexes. In contrast, other studies of high school and collegiate athletes, found that the symptoms exhibited after concussion were sex-specific [13, 14, 19, 20]. Whereas females often report greater declines in cognitive function [13] and a higher number of somatic [14] and neurobehavioral symptoms [14], males have greater odds of reporting confusion [14, 20] and amnesia [14]. To date, there has been no research conducted that investigated potential differences in clinical profile presentation and recovery patterns between male and female collegiate varsity-level athletes following SRC.

The aim of this study was to identify the frequency of each proposed clinical profile, investigate sex differences in clinical profiles, time-to-recovery, and initial symptom severity score after SRC in a large cohort of collegiate student-athletes across the Pacific-12 Conference (Pac-12).

## Methods

### Study Design

Data were obtained from the Pac-12 Concussion Assessment, Research and Education (CARE) Affiliated Program (CAP) and the Pac-12 Health Analytics Program (HAP), which are ongoing prospective cohort studies. The structure and standard procedures of CAP and HAP have been previously described [21, 22]. De-identified data collected as part of the Pac-12 HAP were derived from clinical documentation in a HIPAA Compliant electronic medical record by sports medicine clinicians. Data were de-identified using HIPAA Safe Harbor method for de-identification (45 CFR 164.514). Resulting project data included de-identified records only from student-athletes who provided authorization for secondary research as part of the HAP. Student-athletes were enrolled in this study after providing consent during the period between July 2018 and September 2022.

### Participants

Female and male varsity-level athletes who consented to participate as part of the CAP and HAP studies were included if they sustained a SRC during the study period. Participant sex was self-identified as either male or female.

### Procedures

Prior to the start of each sport’s season, the Sport Concussion Assessment Tool (SCAT5) symptom checklist [23] was administered and defined as “baseline.” Participants also completed a medical history questionnaire from which information on sex, concussion history prior to enrolling at university, and sport played at the time of injury were obtained.

Concussion diagnoses were made by team physicians at each institution using the standardized Concussion in Sport Group definition [24]. The SCAT5 symptom checklist was administered within 48 h of injury, then symptom scores were obtained daily until asymptomatic or return to baseline. A repeat SCAT5 was administered within 24 h of the athlete returning to their baseline and beginning their return-to-play (RTP) protocol. The SCAT5 symptom checklist contains 22 symptoms which are ranked in severity on a likert scale from 0 (no symptom) to 6 (severe symptom). Injury severity was defined as the sum of these symptom scores and could range from a score of 0 to a score of 132. Return-to-play progression was dependent on subjective symptom reporting by student-athletes.

Time to RTP was defined as the number of days an athlete required to complete the entire RTP protocol and to be fully cleared for athletic participation by their team physician. Time-to-recovery was defined as time from injury required for a student-athlete to reach asymptomatic status and complete their final daily SCAT5 symptom evaluation form.

### Clinical Profile Measures

**Fig. 1:**
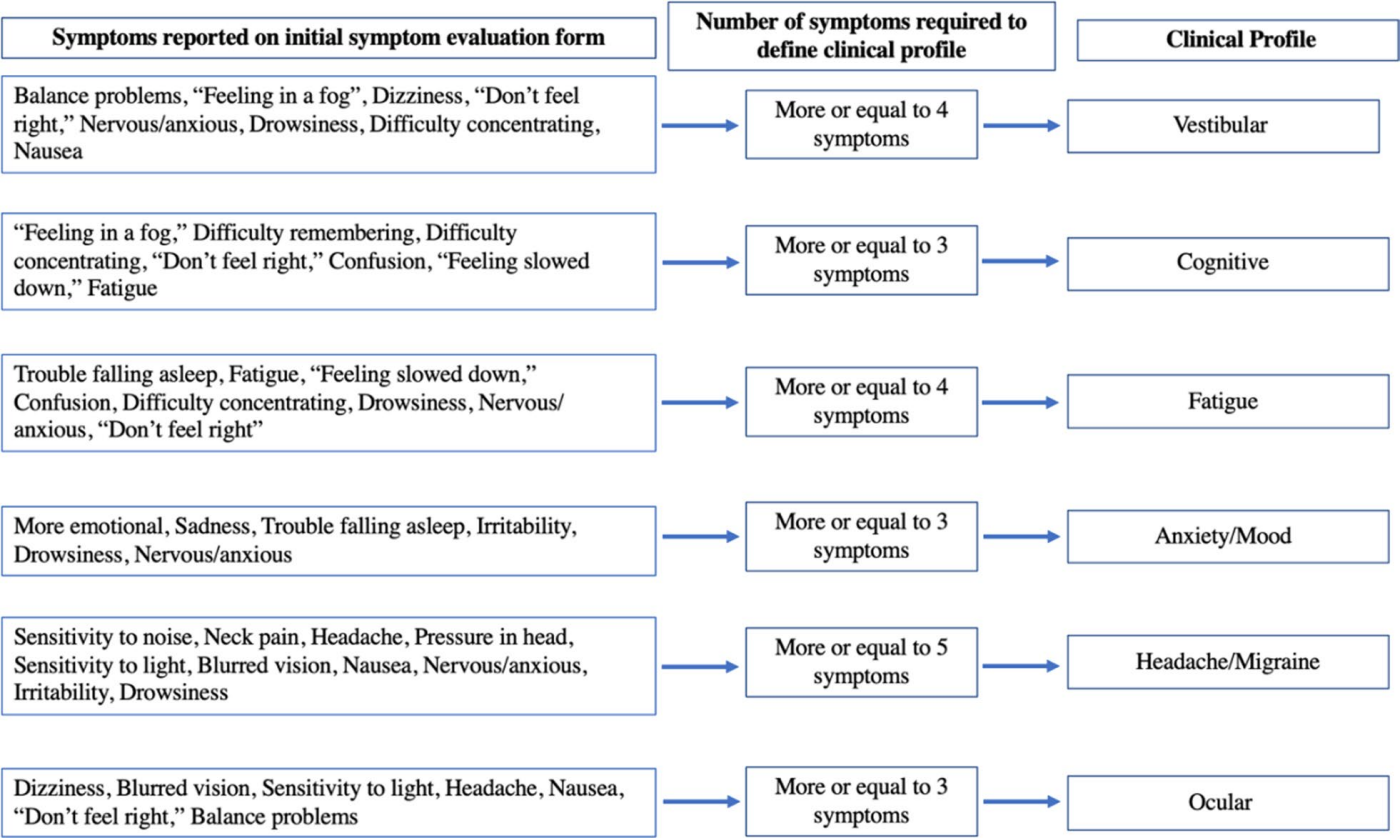
Methods for defining clinical profiles. Each clinical profile is defined by a subset of symptoms as depicted in the first column “symptoms reported on initial symptom evaluation form.” If a participant reported more or equal to 50% of symptoms defining a clinical profile, that participant was determined to have said clinical profile. If a clinical profile was defined by 7 symptoms (e.g., cognitive, anxiety/mood, ocular), then reporting three or more symptoms was required to have the indicated profile

Six clinical profiles were described in this study using the definitions presented by the American Medical Society for Sports Medicine [3]. Clinical profiles are an emerging concept, and although SRC may present with symptoms representing only one clinical profile, it is more likely that SRC will present with symptoms of multiple profiles [3, 6]. Clinical profiles were determined by symptoms reported on the initial and daily SCAT5 Symptom Evaluation Form. To be included, a symptom had to be endorsed with a minimum severity score of “1”, and clinical profiles were not graded by reported symptom severity. Although there are overlapping symptoms among clinical profiles, certain symptoms may be more predominant than others [6]. Therefore, a participant was determined to have a given clinical profile if they reported a predominant number of symptoms, which we defined as ≥ 50% of the symptoms associated with the profile (Fig. 1). Ultimately, profiles were not mutually exclusive and participants could be diagnosed with multiple clinical profiles [25].

### Statistical Analyses

#### Data Formatting

The Pac-12 athletes in our dataset represented 27 different sports. However, many of those sports had relatively few datapoints; for example, only one athlete sustained a concussion while playing golf. Therefore, to meet the cluster size requirements of mixed effects models for maximizing statistical power [26], we collapsed those 27 sports down to 11 total sports groups based on similarity of sport and sample size (Additional file 1: Table S1).

Additionally, of the 579 athletes in our data, 100 sustained concussions either outside of their respective sport (*n* = 82) or it was unclear if the concussion was sport-related or not (*n* = 18). Due to differences in recovery times, return-to-play times, injury severity scores, and number of clinical profiles reported between athletes that sustained concussions from their sport versus in non-sport related or uncertain-origin events (Additional file 1: Fig. S1), all 100 of the non-sport and uncertain-origin concussions were excluded from analysis. This resulted in a final sample size of 479 athletes with known SRC.

#### Model Fitting

We fit a suite of generalized linear mixed models to address the following objectives and questions. For brevity, details of each model are not presented here but are provided in the Additional file 1. First, to determine whether the initial intake values were the best representations for answering our primary questions of interest, we investigated if athletes’ SCAT5 injury severity scores and number of clinical profiles changed over time. Second, we fit models to answer the following questions: (1) did the probability of athletes having a given clinical profile differ between sexes; (2) did initial injury severity scores within a given clinical profile differ between sexes; (3) did time-to-recovery within a given clinical profile differ between sexes; (4) could clinical profiles be predicted by the injury severity scores; and (5) could recovery or return-to-play times be predicted by the number of clinical profiles?

We conducted all statistical analyses in the R statistical computing environment [27]. We fit models using the package glmmTMB [28], and we obtained scaled quantile residuals via simulation to evaluate model fit using Q-Q plots and diagnostic tests (e.g., dispersion and Kolmogorov–Smirnov tests) via the package DHARMa [29]. We produced predicted conditional effects using the package ggeffects [30]. We calculated standardized effect sizes for categorical comparisons (e.g., difference between sexes), and for continuous relationships (e.g., effect of number of clinical profiles on recovery time) we calculated effect sizes as Pearson’s correlation coefficient (*r)* [31]. We based inferences on a combination of coefficient (*β*) estimates, 95% confidence intervals, *p*-values, and effect sizes. The statistical significance determination threshold was *p* < 0.05, whereas biological importance was determined based on effect size values with the following scale (Cohen, 1988; Nakagawa & Cuthill, 2007): A) nominal magnitude: *d* < 0.2 or *r* < 0.7; B) small magnitude: 0.2 ≤ *d* < 0.5 or 0.7 ≤ *r* < 0.8; C) medium magnitude: 0.5 ≤ *d* < 0.8 or 0.8 ≤ *r* < 0.9; D) large magnitude: *d* ≥ 0.8 or *r* ≥ 0.9.

## Results

### Sample Characteristics

A total of 479 concussion incidents met inclusion criteria. Male athletes were slightly more represented than female athletes (Table 1), but the sex ratio was not statistically different from 1:1 (*χ*^2^ = 0.08; *p* = 0.78). From the initial SCAT5, most participants reported symptoms associated with ocular and cognitive profiles, followed by fatigue, vestibular, headache/migraine disorders, and lastly, anxiety/moodiness (Table 1).

**Table 1:** Descriptive characteristics of Pac-12 athletes’ sex and reported clinical profiles during the initial intake evaluations

Sex	*N* (%)		
Female	275 (47.5)		
Male	304 (52.5)		

Clinical profile	*N* (%) *	Female (%) **	Male (%) **
Vestibular	324 (56)	149 (46)	175 (54)
Cognitive	383 (66.1)	175 (45.7)	208 (54.3)
Fatigue	330 (57)	151 (45.8)	179 (54.2)
Anxiety/Moodiness	206 (35.6)	99 (48.1)	107 (51.9)
Headache/Migraine	310 (53.5)	142 (45.8)	168 (54.2)
Ocular	395 (68.2)	180 (45.6)	215 (54.4)

*Percent of total number of participants

**Percent of total participants who reported specified clinical profile

### Temporal Trends in Injury Severity Scores and Number of Clinical Profiles

Among all considered models, those with zero-inflated negative-binomial error distributions and nonlinear effects of time (days post-injury) best fit the repeated measures SCAT5 injury severity scores and number of clinical profiles. A very strong nonlinear effect of time on both injury severity scores and number of clinical profiles existed (*β*_Days_ = − 9.95–4.41; *p* = 0.0001–0.03). The most rapid decline in injury severity scores and number of clinical profiles occurred within the first 14 days post-injury (Severity_0–14 Days_: 92% decline; Profiles_0–14 Days_: 69% decline), and the initial intake values for both injury severity scores and number of clinical profiles were the highest (Additional file 1: Fig. S2A–B). Therefore, we used the initial intake values of both responses in all subsequent analyses, unless specified otherwise below. Notably, the models’ random intercepts specification of athletes nested within their sports groupings accounted for ~ 52% of the variance explained by each model, and the overall variance explained by the injury severity scores model and number of clinical profiles model was 85% and 77%, respectively, based on coefficient of determination values [33]. For comparison, similar models that instead modeled time as linear rather than nonlinear explained 61% and 72% of the variance in injury severity scores and number of clinical profiles, respectively, demonstrating superior fit of models with a nonlinear time effect.

### Sex Differences in Clinical Profiles, Injury Severity Scores, and Time-to-Recovery

**Fig. 2:**
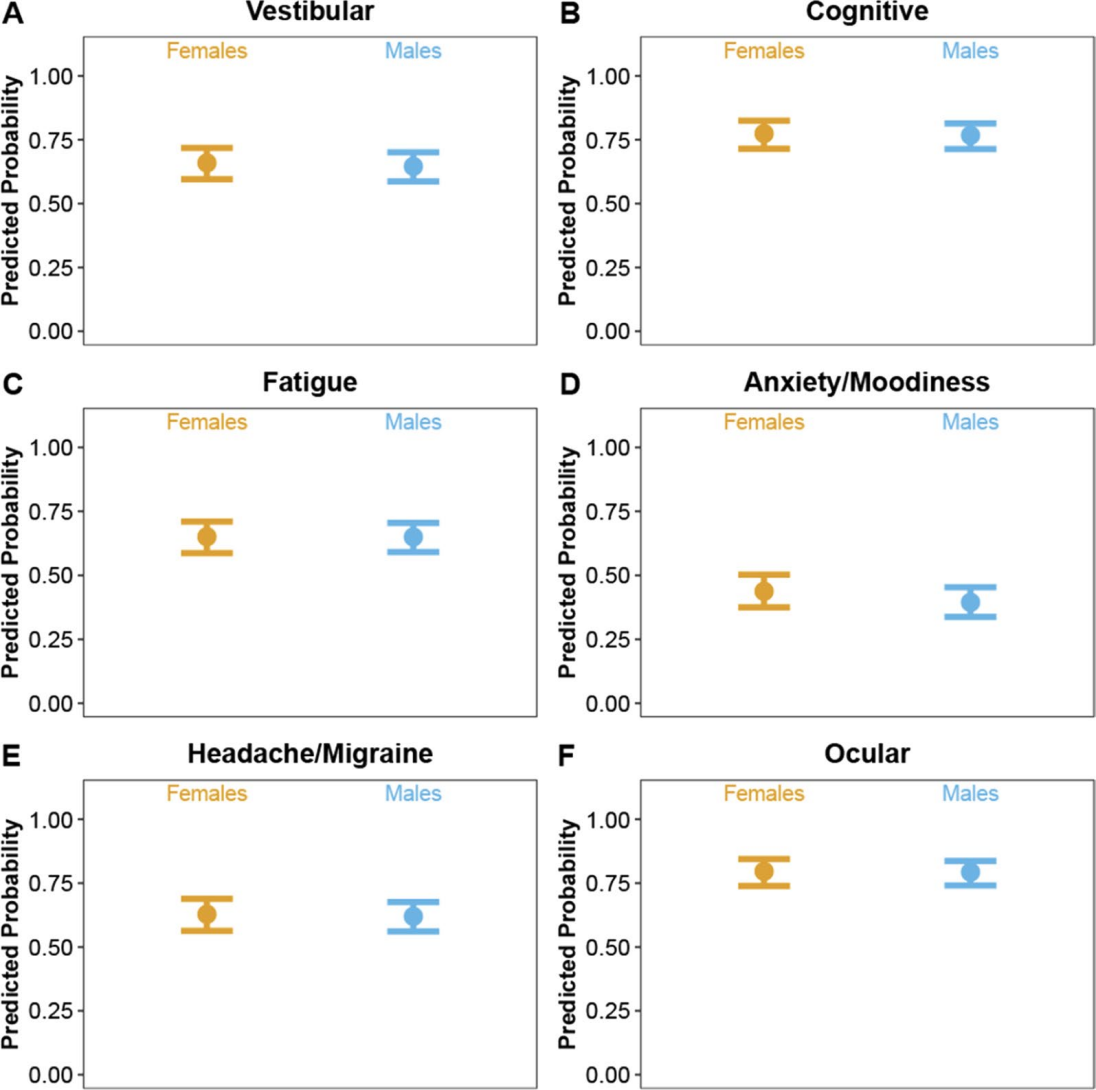
Predicted probability point estimates (solid dots) and 95% confidence intervals (error bars) of male and female student-athletes reporting **A** vestibular, **B** cognitive, **C** fatigue, **D** anxiety/moodiness, **E** headache/migraine, and **F** ocular clinical profiles during acute concussion (based on symptom scores from initial Scat5 form)

**Fig. 3:**
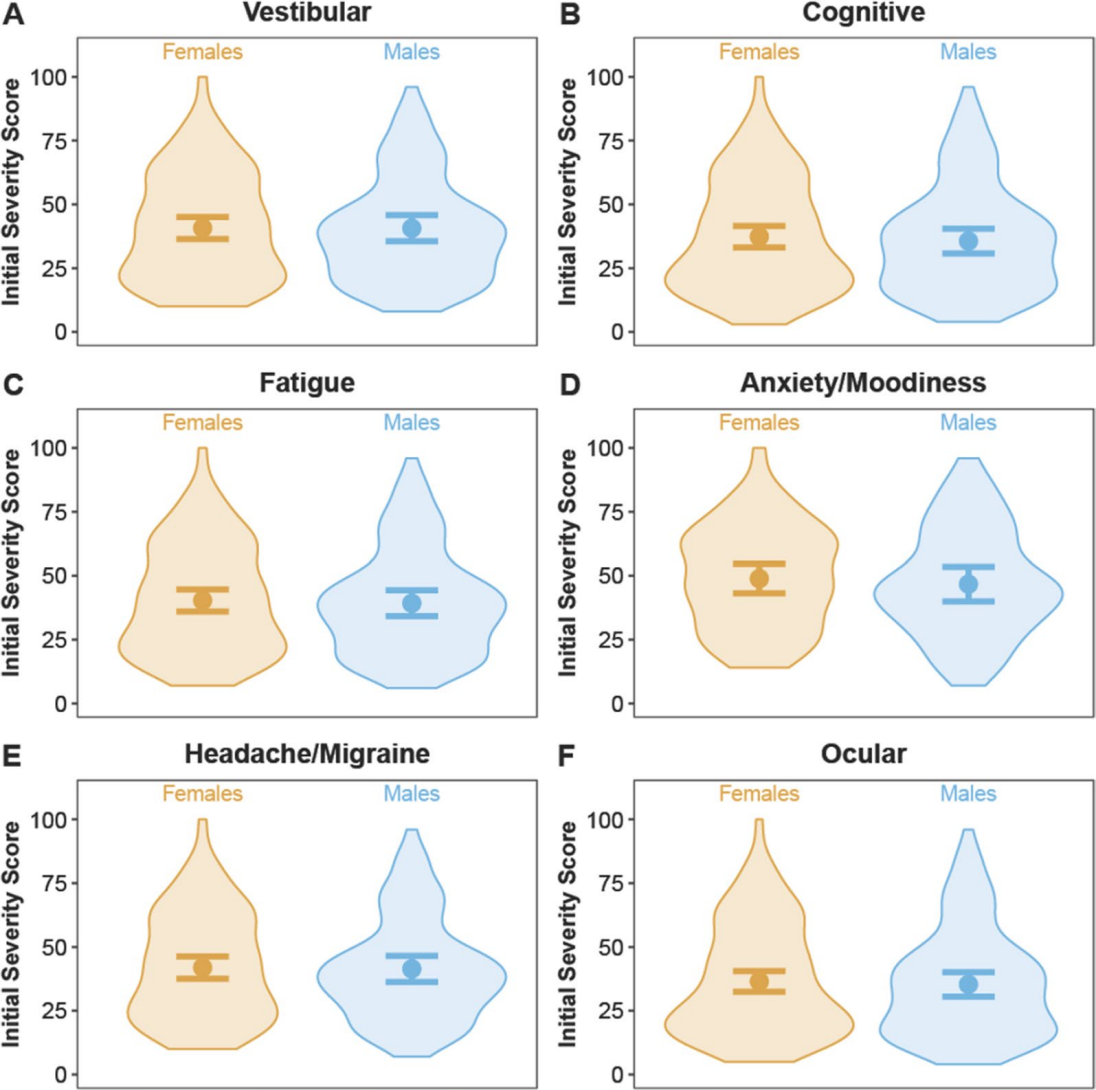
Model-predicted mean conditional effects point estimates (solid dots) and their 95% confidence intervals (error bars) of sex-specific initial severity scores for student-athletes who sustained a concussion and had the** A** vestibular, **B** cognitive, **C** fatigue, **D** anxiety/moodiness, **E** headache/migraine, and **F** ocular clinical profiles at acute concussion (based on symptom scores from initial Scat5 form). Background violin plots depict the distributions of the raw data

**Fig. 4:**
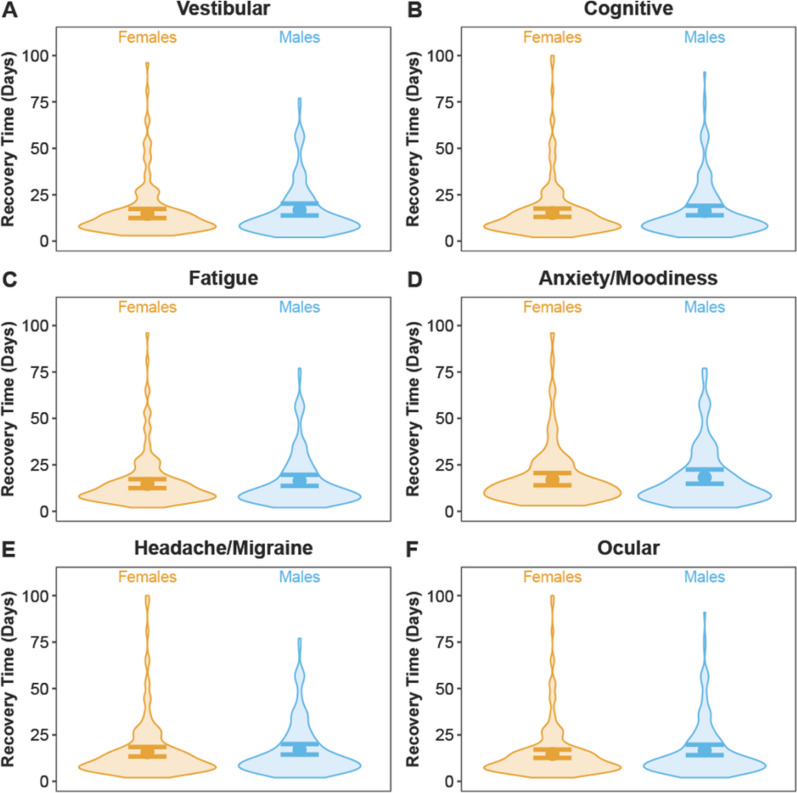
Model-predicted mean conditional effects point estimates (solid dots) and their 95% confidence intervals (error bars) of sex-specific recovery times (days) for student-athletes who sustained a concussion and had the **A** vestibular, **B** cognitive, **C** fatigue, **D** anxiety/moodiness, **E** headache/migraine, and **F** ocular clinical profiles at acute concussion (based on symptom scores from initial Scat5 form). Background violin plots depict the distributions of the raw data

Males and females did not have different probabilities of initially presenting a given clinical profile, different initial injury severity scores within a profile, or different recovery times within a profile. However, both sexes had 0.50–0.75 probability of having the vestibular, fatigue, and headache/migraine profiles, and > 0.75 probability of having the cognitive and ocular profiles (Fig. 2A–F). Mean initial injury severity scores within each profile ranged from 30 to 50, indicating that, on average, most athletes reported similar injury severity, regardless of which clinical profiles they presented with (Fig. 3A–F). Mean recovery times based on clinical profile presentation ranged from 13 to 18 days for both sexes (Fig. 4A–F).

### Predicting the Number of Clinical Profiles, Recovery Times, and Return-to-Play Times

**Fig. 5:**
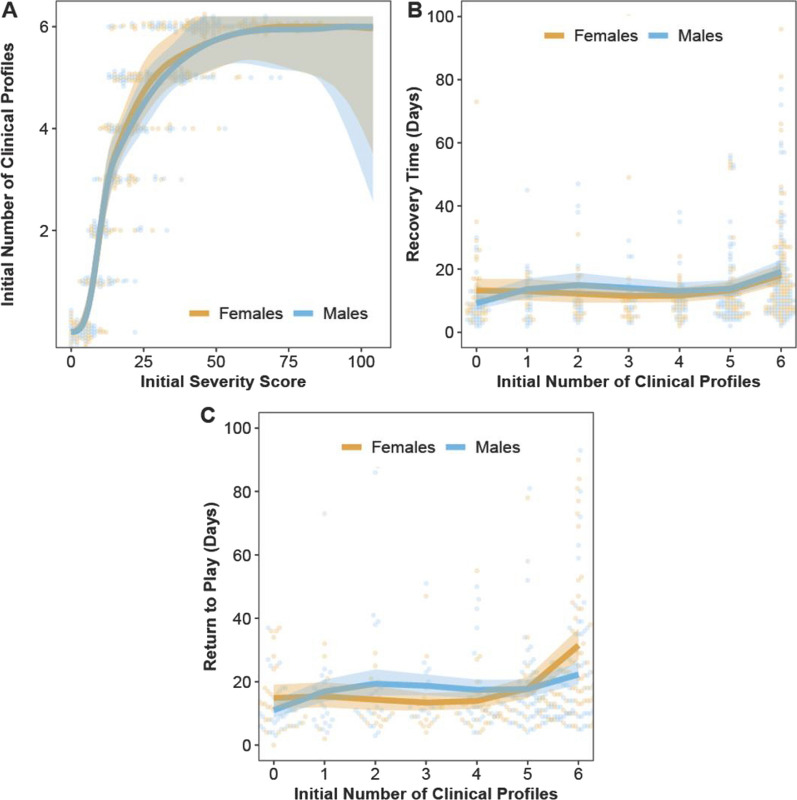
Model-predicted mean conditional effects point estimates (solid lines) and their 95% confidence intervals (ribbons) of the **A** initial number of clinical profiles predicted by initial injury severity scores, **B** recovery times (days) predicted by initial number of clinical profiles, and **C** return-to-play times (days) predicted by initial number of clinical profiles, for male and female student-athletes. Background dots denote the raw data points

Initial injury severity scores were very strong, positive, nonlinear predictors of the initial number of clinical profiles (*β*_Scores_ = 6.44–7.66; *p* < 0.0001), which did not differ between sexes (*β*_Sex:Scores_ = − 0.20–0.04; *p* = 0.91–0.99; Fig. 5). This relationship mirrored the logistic growth function, such that, on average, initial injury severity scores < 10 were predicted to have 0–1 clinical profiles, scores ≥ 10 but ≤ 43 were predicted to have 2–5 profiles, and scores > 43 were predicted to have all 6 clinical profiles (Fig. 5A). This model explained 92% of the variance in the initial number of clinical profiles.

Initial number of clinical profiles was a relatively weak but positive nonlinear predictor of recovery times (*β*_Profiles_ = 0.31; *p* = 0.03). Mean recovery times differed between sexes only when athletes presented 0 clinical profiles, such that females were predicted to have recovery times that were 4 days longer than males; although, the effect size was small (*p* = 0.04, *d* = 0.16; Fig. 5B). Across all numbers of clinical profiles, the estimated mean recovery times were 13 (95% CI 10–16) and 14 (95% CI 11–17) days post-injury for females and males, respectively. This model explained 11% of the variance in recovery times.

Relative to the recovery times model, the initial number of clinical profiles was a positive nonlinear predictor of return-to-play times (*β*_Profiles_ = 0.75; *p* < 0.0001). Mean return-to-play times significantly differed between sexes only when athletes presented 3 and 6 clinical profiles, but the effects differed for each of those profile numbers. With 3 clinical profiles, males were predicted to have return-to-play times that were 6 days longer than females (*p* = 0.01, *d* = 0.15), whereas with 6 clinical profiles, females were predicted to have return-to-play times that were 9 days longer than males (*p* = 0.002, *d* = 0.16; Fig. 5C). Across all numbers of clinical profiles, the estimated mean return-to-play times were 17 (95% CI: 14–20) and 18 (95% CI: 14–22) days post-injury for females and males, respectively. This model explained 14% of the variance in return-to-play times.

## Discussion

Our study examined clinical profile presentation, time-to-recovery, and time to return-to-play, in male and female collegiate athletes in the Pac-12 after SRC. At concussion diagnosis, males and females had the highest probabilities of reporting cognitive and ocular profiles. In a study of young-adults with similar age (19.11 years), migraine and anxiety mood disorder profiles were the most commonly reported post-concussion [6]. Participants in this study were evaluated at 15.3 days post-concussion, whereas student-athletes across the Pac-12 in our study were evaluated immediately or within 48 h of injury, possibly contributing to differences in reported profiles throughout recovery [34]. No evidence was found in support of either sex having a different probability of initial presentation of a given clinical profile, initial severity score, or difference in time-to-recovery. Similarly, research of adolescent and college-aged athletes have found no sex-based differences in neurocognitive performance or recovery patterns after concussion [17, 18]. However, we found that for both sexes, the most rapid decline in injury severity scores and number of clinical profiles reported among Pac-12 athletes occurred within the first 14 days post-injury, with recovery occurring between 14 and 18 days, on average. This mean time-to-recovery was slightly longer than the purported timeframe of 2 weeks [24], though not substantially different.

Nevertheless, we found considerable evidence that self-reported symptoms resolved, and therefore recovery occurred, in a nonlinear pattern across time. Although both males and females exhibited a sharp decline in symptom severity within 3 weeks post-injury, some athletes remained symptomatic even 2 months post-injury. These findings are similar to that of the NCAA-DoD CARE Consortium, which found that both males and females showed steady symptom reduction during the first 3 weeks after injury, with males being more likely than females to be asymptomatic by the fourth week [35]. The number of clinical profiles reported per clinical visit also declined in a nonlinear pattern over time; however, as supported by previous studies of collegiate varsity and non-varsity athletes, females were more likely than males to report persistent symptoms after concussion [11, 36].

Recovery times for both sexes within each clinical profile were severely right skewed with long tails, which was most likely caused by some athletes who exhibited multiple clinical profiles and required much longer than average times to reach asymptomatic status. Research in adolescent and college varsity-level athletes directly addressing differences in post-concussion time-to-recovery between sexes is limited and has produced conflicting results [5, 17]. Some Pac-12 athletes reported having 0 clinical profiles, meaning that, although symptomatic, the individual did not report a large enough number of certain symptoms to fall under a specific symptom profile. Notably, females required longer time-to-recovery than males only when zero clinical profiles were reported, otherwise no significant sex differences in time-to-recovery were found. This suggests that females may experience a broader range of symptoms but a fewer number of symptoms within a given profile than males, but as the number of symptoms increases, males and females exhibit similar recovery patterns. However, at 60–80 days after injury, some females and males did report an increase in symptom severity. It has been postulated that, compared to males, females may report persistent symptoms [11, 12, 37] after concussion due to biological differences [38] or a greater willingness to report symptoms associated with concussion [39].

Although the number of initial profiles was weakly associated with time-to-recovery and was a slightly stronger predictor of return-to-play time, neither model explained much variance in recovery or in return-to-play times (< 15%). In contrast, initial severity score was a strong positive predictor of the initial number of clinical profiles reported following concussion, and this hierarchical nonlinear model explained most of the variance in number of clinical profiles (92%). Therefore, although the number of initial profiles was not strongly associated with differences in time-to-recovery or time to return-to-play, initial severity score did explain differences in number of clinical profiles reported at acute injury.

Previous research in collegiate athletes has found that greater total symptom severity in the acute post-injury period is associated with increased white-matter abnormalities [40]. Those findings combined with our results might suggest that, as concussion symptom severity increases, there is a greater likelihood of widespread neurocognitive dysfunction, which could cause an athlete to experience a variety of symptoms associated with concussion. The number of initial clinical profiles reported was a very weak predictor of time to asymptomatic but was a slightly stronger predictor of return-to-play times. We found that at 3 clinical profiles, males required 6 more days to RTP than females, which conflicts with an NCAA study that found male athletes reported RTP duration that was abbreviated by 1 day compared to female athletes [35]. However, among athletes who reported 6 clinical profiles, we found that females required 9 more days to RTP than males. This difference in time to return-to-play at varying concussion severity may suggest that females may maintain better self-reporting behaviors throughout return-to-play progression than males [41].

### Limitations

Concussion is a clinical diagnosis without an objective gold standard, which may have caused variation in how concussion was diagnosed and managed at the 12 participating institutes. However, a Pac-12 Conference-wide standard protocol [21] was enforced at all institutions, with oversight from research personnel, to manage and minimize variation in concussion reporting and data collection. Time-to-recovery was defined by the final SCAT5 exam date; therefore, recovery may have been impacted by diligence of clinical care. While possible, concussion recovery reported in this study is similar to findings from other studies conducted in collegiate athletes, in which time to asymptomatic [35] and completion of RTP protocol [35, 42] sometimes required ≥ 4 weeks. Therefore, our investigation most likely shows an accurate representation of time-to-recovery and time to return-to-play among collegiate athletes. Lastly, we failed to control for previous neurological or psychiatric problems, which have been associated with prolonged recoveries [43].

## Conclusions

This study increases understanding of the variability in acute and long-term concussion symptom presentation using defined clinical profiles. We found that both males and females reported a higher probability of developing cognitive and ocular symptoms following an SRC as compared to all other clinical profiles. Diagnosis of concussion has been historically difficult because of the heterogenous nature of the injury and its sequelae; thus, these findings may aid in the development of cognitive and ocular symptom assessments for concussion diagnosis. Time-to-recovery and time to return-to-play were consistent with existing literature on collegiate athletes. However, the number of clinical profiles reported, and initial symptom severity were associated with prolonged SRC recovery in athletes, whereas clinical profile type was not. Sex was also not a strong predictor for time-to-recovery or time to return-to-play. Ultimately, although athletes may experience clusters of symptoms associated with proposed clinical profiles, the immense overlap between symptoms associated with each profile may impede the use of these profiles for diagnostics and treatment. Our results may be used to add to the understanding of conceptualized clinical profiles, and further inform targeted symptom assessments and recovery strategies.

### Supplementary Information

**Additional file 1.** provides one table describing sports groups represented in the data, and figures illustrating the differences in injury characteristics of non-sport and sport-related concussion, as well as sex differences in severity score and number of clinical profiles daily after injury. Additional details of the methods for statistical analyses is also included.

## Data Availability

Data are available upon reasonable request.

## Abbreviations

SRC: Sport-related concussion
Pac-12: Pacific-12 Conference
CARE: Pac-12 Concussion Assessment, Research and Education
CAP: Pac-12 CARE-Affiliated Program
HAP: Pac-12 Health Analytics Program
SCAT5: Sport Concussion Assessment Tool
RTP: Return-to-play
