# Supplementary material for: Nominal Differences in Acute Symptom Presentation and Recovery Duration of Sport-Related Concussion Between Male and Female Collegiate Athletes in the PAC-12
Source: Sports Med Open. 2024 Apr 2;10:31. doi: 10.1186/s40798-024-00699-4 (PMC10987417; doi:10.1186/s40798-024-00699-4)
Supplement: Supplementary file 1 — Additional file 1. provides one table describing sports groups represented in the data, and figures illustrating the differences in injury characteristics of non-sport and sport-related concussion, as well as sex differences in severity score and number of clinical profiles daily after injury. Additional details of the methods for statistical analyses is also included. [file 40798_2024_699_MOESM1_ESM.pdf]

Nominal differences in acute symptom presentation and recovery duration between male and female collegiate athletes in the PAC-12

Niki A. Konstantinides[1]*, Sean M. Murphy[2], Bridget M. Whelan[3], Kimberly G Harmon[3], Sourav K Poddar[4], Theresa D. Hernández[5], Rachel K. Rowe[1]

[1] Department of Integrative Physiology, University of Colorado – Boulder, Boulder, CO, USA

[2] Cumberland Biological and Ecological Researchers, Longmont, CO, USA

[3] Family Medicine, University of Washington, Seattle, WA, USA

[4] Department of Family Medicine, University of Colorado- Anschutz Medical Campus, Aurora, CO USA

[5]Psychology and Neuroscience (CU Boulder), Physical Medicine and Rehabilitation (CU Anschutz School of Medicine), CO, USA

*Corresponding Author

Niki Konstantinides, Ph.D.

Department of Integrative Physiology

University of Colorado Boulder, UCB 354

Boulder, CO 80309

niki.konstantinides@colorado.edu

SUPPLEMENTAL INFORMATION

Table S1. Pac-12 sports represented in the data, the sport groups that individual sports were combined into for inclusion as random intercepts in the mixed models, and the corresponding sample sizes of athletes (*n*) after removing the 100 athletes with non-sport and uncertain-origin concussions.

Sport	*n*	Sport Group	*n*
Baseball	13	BaseSoftball	45
Softball	32		
Basketball	34	Basketball	40
Men's Basketball	2		
Women's Basketball	4		
Volleyball	48	Volleyball	55
Beach Volleyball	7		
Track and Field	3	CCTrack	6
Cross Country	1		
Cross Country & Track	2		
Swimming	5	SwimDive	13
Men's Swimming	1		
Women's Swimming	1		
Diving	4		
Women's Diving	2		
Football	176	Football	176
Gymnastics	13	Gymnastics	13
Lacrosse	16	Lacrosse	16
Soccer	50	Soccer	50
Water Polo	12	Water Polo	12
Golf	1	OtherSports	53
Rowing	6		
Skiing	4		
Tennis	3		
Wrestling	6		
Unique Sports	31		
Unknown	2		

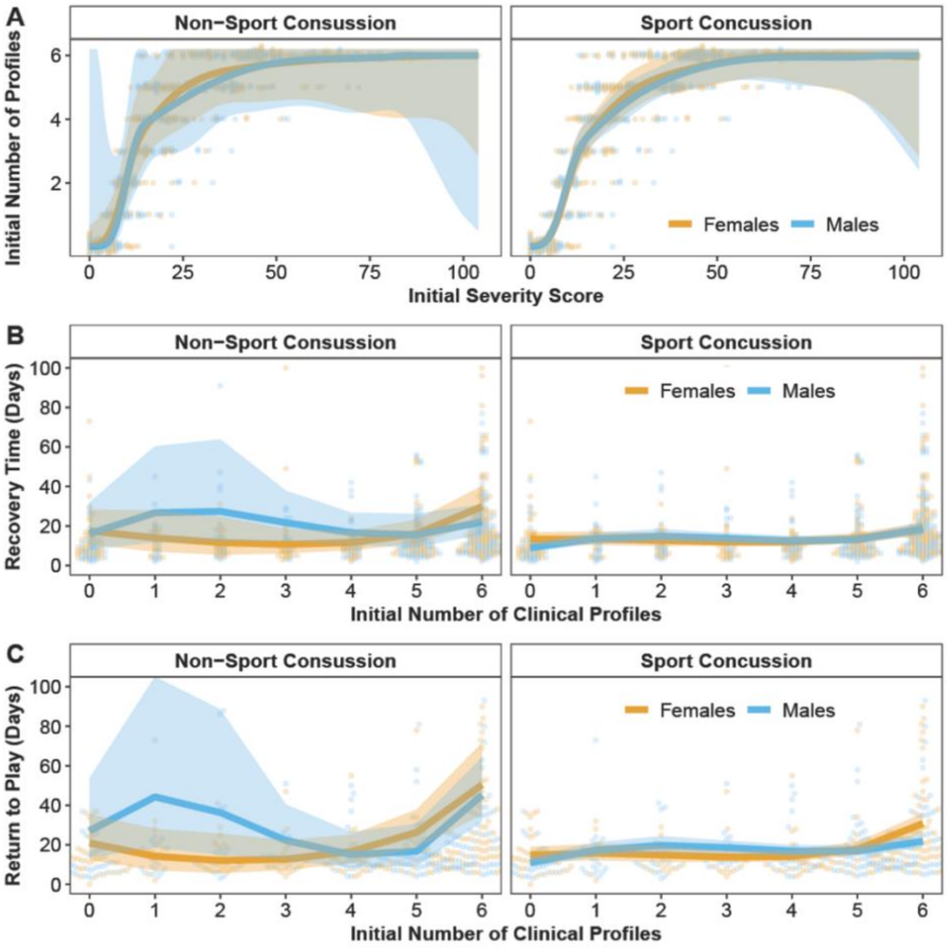

Figure S1: Predicted conditional effects from generalized linear mixed models that tested for differences between non-sport and sport-related concussions in **(A)** the initial number of clinical profiles by initial severity scores, **(B)** the recovery times by initial number of clinical profiles, and **(C)** the return-to-play times by initial number of clinical profiles. Solid lines, shaded regions, and background points denote the sex-specific mean conditional effects, their 95% confidence intervals, and the raw data, respectively.

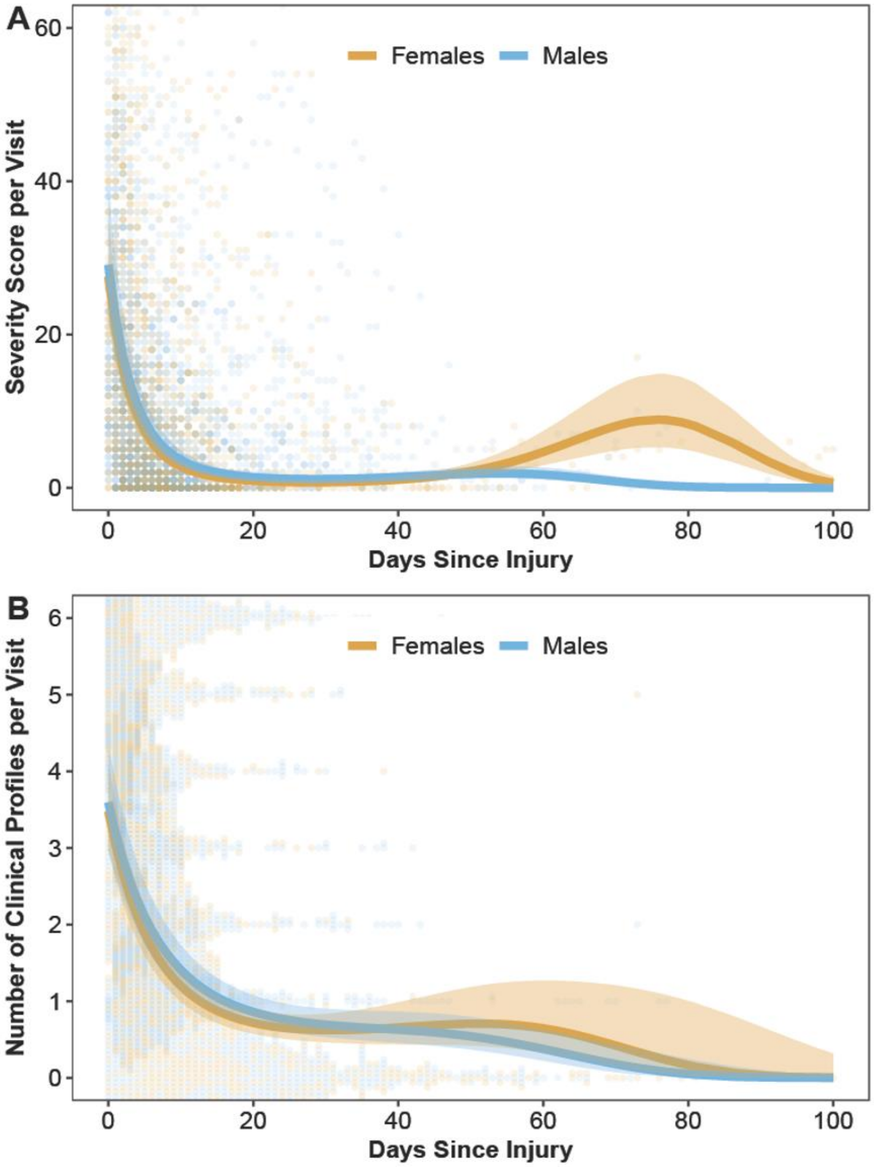

Figure S2: Model-predicted mean conditional effects point estimates (solid lines) and their 95% confidence intervals (ribbons) of the **(A)** initial symptom severity score from the Scat5 and **(B)** number of clinical profiles reported per visit. Background dots denote the raw data points.

Details of Statistical Analysis Methods

Temporal trends in injury severity scores and number of clinical profiles – We investigated if athletes' SCAT5 injury severity scores and number of clinical profiles changed over time to determine whether the initial intake values were the best representations for answering our primary questions of interest. We first conducted exploratory analyses to determine whether Poisson or negative-binomial distributions, or zero-inflated versions of those distributions, best described the initial injury severity scores and number of clinical profiles (40–42). To do this, we fit models with said distributions and then conducted information-theoretic model selection using Akaike's Information Criterion (AIC) to identify the model with the best distribution for describing the data (43). Using the most supported error distribution, we then fit models that included either a linear or nonlinear effect of time (days post-injury) that interacted with athlete sex to determine if linear or nonlinear temporal trends best described the data. We used basis splines with 3 degrees of freedom to model the nonlinear time effect (44). In all those models, we included random intercepts for athletes' injury identification numbers nested within the sport group that each athlete played; this random effects specification accounted for the correlation among repeated measures on individual athletes over time as well as hierarchical clustering of athletes within individual sports groups. We again used AIC to determine whether the linear or nonlinear temporal models best fit the injury severity scores and number of clinical profiles.

Sex differences in injury severity scores, clinical profiles, and recovery times – Previous studies found differential outcomes and recovery times following concussion in male and female athletes, so we suspected that athletes' injury severity scores and clinical profiles may differ between the sexes (13,45). Therefore, we investigated if the probability of athletes having a given clinical profile differed between sexes, if initial injury severity scores within a given clinical profile

differed between sexes, and if recovery times within a given clinical profile differed between sexes. For the clinical profile response models, we specified binomial error distributions because athletes could either have (1) or not have (0) a given profile. For the initial injury severity score and recovery time response models, we specified negative-binomial error distributions because the severity scores were effectively total counts (*i.e.,* sums) and the recovery times were counts of the number of days post-concussion, both of which were overdispersed. We used only the initial intake values for the clinical profile and injury severity score response models, and only the final time values that denoted recovery for the recovery time models, so we did not include random intercepts for athletes' injury identification numbers because there were no repeated measures; however, we did include random intercepts for the sport group that each athlete played when the concussion occurred.

Predicting the number of clinical profiles, recovery times, and return-to-play times – From a clinical perspective, we were interested in whether the clinical profiles could be predicted by the injury severity scores and, as an extension, whether the recovery or return-to-play times could be predicted by the number of clinical profiles. Therefore, we fit three separate models to test these predictions: 1) a number of clinical profiles response model with a fixed effects interaction between sex and initial injury severity score; 2) a recovery time response model with a fixed effects interaction between sex and initial number of clinical profiles; and 3) a return-to-play time response model with a fixed effects interaction between sex and initial number of clinical profiles. Because we subset the data to the initial/intake clinical profiles and final recovery and return-to-play times, we did not include random intercepts for athlete's injury identification numbers; however, we did include random intercepts for the sport group that each athlete played when the concussion occurred. For the initial number of clinical profiles response models, we specified

Poisson error distributions because there was no evidence of under or overdispersion. For the recovery and return-to-play times response models, we specified negative-binomial error distributions because both were counts of the number of days and were overdispersed. We included nonlinear effects of initial injury severity scores and initial numbers of clinical profiles in those models, which we modeled using basis splines with 3–5 degrees of freedom.
